# Multiple Adaptive Neuro-Fuzzy Inference System with Automatic Features Extraction Algorithm for Cervical Cancer Recognition

**DOI:** 10.1155/2014/181245

**Published:** 2014-02-23

**Authors:** Mohammad Subhi Al-batah, Nor Ashidi Mat Isa, Mohammad Fadel Klaib, Mohammed Azmi Al-Betar

**Affiliations:** ^1^Department of Computer Science and Software Engineering, Faculty of Science and Information Technology, Jadara University, P.O. Box 733, Irbid, Jordan; ^2^Imaging and Computational Intelligence (ICI) Group, School of Electrical & Electronic Engineering, Universiti Sains Malaysia, Engineering Campus, 14300 Nibong Tebal, Penang, Malaysia; ^3^Software Engineering Department, College of Computer Science & Engineering, Taibah University P.O. Box 344, Madinah 30001, Saudi Arabia; ^4^Department of Information Technology, Al-Huson University College, Al-Balqa Applied University, P.O. Box 50, Jordan; ^5^School of Computer Sciences, Universiti Sains Malaysia, 11800 Penang, Malaysia

## Abstract

To date, cancer of uterine cervix is still a leading cause of cancer-related deaths in women worldwide. The current methods (i.e., Pap smear and liquid-based cytology (LBC)) to screen for cervical cancer are time-consuming and dependent on the skill of the cytopathologist and thus are rather subjective. Therefore, this paper presents an intelligent computer vision system to assist pathologists in overcoming these problems and, consequently, produce more accurate results. The developed system consists of two stages. In the first stage, the automatic features extraction (AFE) algorithm is performed. In the second stage, a neuro-fuzzy model called multiple adaptive neuro-fuzzy inference system (MANFIS) is proposed for recognition process. The MANFIS contains a set of ANFIS models which are arranged in parallel combination to produce a model with multi-input-multioutput structure. The system is capable of classifying cervical cell image into three groups, namely, normal, low-grade squamous intraepithelial lesion (LSIL) and high-grade squamous intraepithelial lesion (HSIL). The experimental results prove the capability of the AFE algorithm to be as effective as the manual extraction by human experts, while the proposed MANFIS produces a good classification performance with 94.2% accuracy.

## 1. Introduction

Cervical cancer is a malignant disease that develops in the cells of the cervix or the neck of the uterus [1]. It is considered as the second most common form of cancer in women worldwide, ranked after breast cancer [2]. It causes loss of productive life in women both due to early death as well as prolonged disability. The primary reason is lack of awareness of the disease and access to screening and health services [3]. According to survey, one woman dies every seven minutes of cervical cancer and by the year 2025, it is estimated to be one death in every five minutes [4]. However, in most cases, cervical cancer takes many years to develop from normal to advanced stage [5, 6]. Hence, the incidence and mortality related to this disease can be significantly reduced through early detection and proper treatment [7, 8].

There are wide varieties of screening techniques for cervical cancer [9–13]. Papanicolau smear test is a well-known screening method for detecting abnormalities of the cervix cells [14]. Due to scarce number of skilled and experienced cytologists, the screening procedure becomes time consuming and highly prone to human errors that leads to inaccurate and inconsistent diagnosis [15]. This condition increases the risk that patients who get human papillomavirus (HPV) infection will not be detected and become HPV carriers. These factors can hide and obscure the important cervical cells morphologies, thus increasing the rate of false diagnosis rate [16].

Nowadays, computer-aided screening or diagnostic system based on artificial intelligence (AI) could be a promising tool to produce a more accurate and faster screening and/or diagnostic result for cancer patients. A considerable number of researches were carried out specifically with the attempts to automate both the Pap smear and the LBC classification based on AI [17–20]. The classification requires cervical cell features to be extracted manually by human expert from the Pap smear and the LBC image as the input data [21, 22]. The automated classification system may not only reduce the time required for sample classification, but also reduce the misclassification of sample due to eye fatigue or human error [23, 24]. Therefore, this work presents automatic recognition method for cervical cancer images that contains single cervical cell. The proposed method comprises feature extraction and classification. The feature extraction is a three-step process with clustering, centroid computation, and segmentation. The classification is based on neuro-fuzzy inference.

## 2. The Proposed System

**Figure 1:**
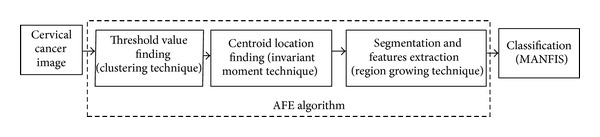
Cervical cancer computer vision system stages.

An intelligent computer-vision system is proposed to enable automatic recognition of the cancerous cells. Firstly, the images are captured from the slides by using the AutoCapture system [25]. A total of 500 single cell images were selected from images captured for this study. Then, the automatic features extraction (AFE) algorithm is implemented to extract the features from the images [26]. The AFE algorithm involves moving *k*-mean (MKM) algorithm as clustering algorithm, seed-based region growing (SBRG) algorithm for the segmentation, and modified SBRG algorithm for features extraction process. The classification is developed using multiple adaptive neuro-fuzzy inference system (MANFIS).  Figure 1 summarizes the flow of process of the proposed system.

## 3. Automatic Features Extraction Algorithm

### 3.1. Threshold Value Finding

The *k*-means and fuzzy *c*-means clustering algorithms are commonly used in medical image segmentation [27, 28]. Many researchers have proved that moving *k*-means (MKM) algorithm produces better features extraction performance of cervical cancer image as compared to *k*-means and fuzzy *c*-means clustering algorithms [29, 30]. In this study, MKM clustering algorithm is used to segment the image into three regions; that is, nucleus, cytoplasm, and background. Thus, the number of clusters is set to three (*n*_*c*_ = 3).

**Figure 2:**
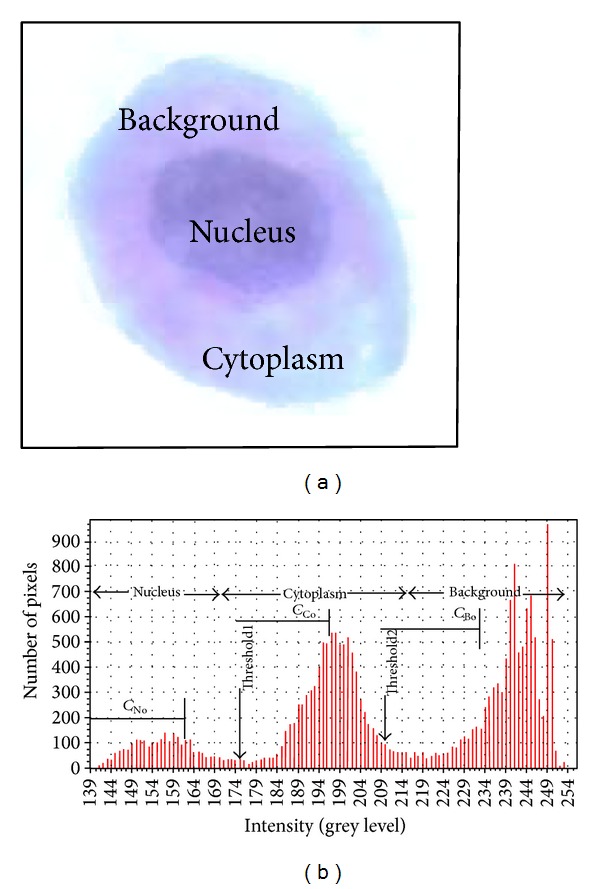
An example of image histogram; divided into 3 regions, nucleus, cytoplasm, and cell background.

The features extraction process begins with segmentation process that partitions the cervical cell into three main regions: nucleus, cytoplasm, and background of the cell (Figure 2). Grey level histogram of the cervical cell image is analyzed to obtain the initial centre of each region: *C*_*No*_, *C*_*Co*_, and *C*_*Bo*_, for nucleus, cytoplasm, and background, respectively, as illustrated in Figure 2. Each region has a different range of grey level that starts with nucleus which has the lowest value, followed by cytoplasm and background. The MKM clustering algorithm is then used to determine the final value of each centre that is referred to as *C*_*N*_, *C*_*C*_, and *C*_*B*_ for nucleus, cytoplasm, and background, respectively. Based on the Euclidean distance concept, the threshold value, *β*_*NC*_ and *β*_*CB*_, is calculated based on

(1) $$\begin{array}{c} \beta_{NC}=\frac{\left(C_{N}+C_{C}\right)}{2}{,\;\;\beta }_{CB}=\frac{\left(C_{C}+C_{B}\right)}{2}, \end{array}$$

where *β*_*NC*_ is the threshold value to differentiate the nucleus-cytoplasm area and *β*_*CB*_ is the threshold value to differentiate the cytoplasm-background area.

**Figure 3:**
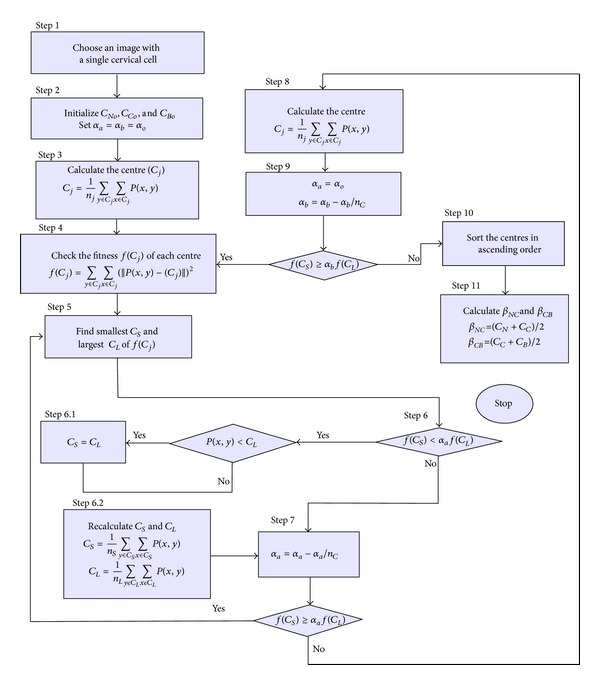
Flowchart summarising the method used by moving *k*-means clustering.

To discuss the MKM algorithm, consider  *P*(*x*, *y*), pixels of one digital image with resolution of *M* × *N*, where *x* = 0, 1, 2, 3,…, *M* − 1 and *y* = 0, 1, 2, 3,…, *N* − 1. The image is clustered into  *n*_*c*_ clusters (here, *n*_*c*_ = 3 to represent nucleus, cytoplasm, and background areas) and has *C*_*j*_ centres (the centres are *C*_*N*_, *C*_*C*_, and *C*_*B*_ for nucleus, cytoplasm, and background of the cervical cell, resp.). The MKM algorithm that is used for threshold values finding on cervical cell image is described and flowcharted in Figure 3.

### 3.2. Centroid Location Finding

The centroid, or usually known as seed of the cervical cell image, is located by using the invariant moment technique [31]. The seed is used as a starting point for segmentation and features extraction process [32]. The centroid of cervical cell image is located inside the nucleus of the cell. Thus, a process to eliminate the cytoplasm and the background region is needed before the process of obtaining the centroid is applied. This process is called subclustering process [33].

**Figure 4:**
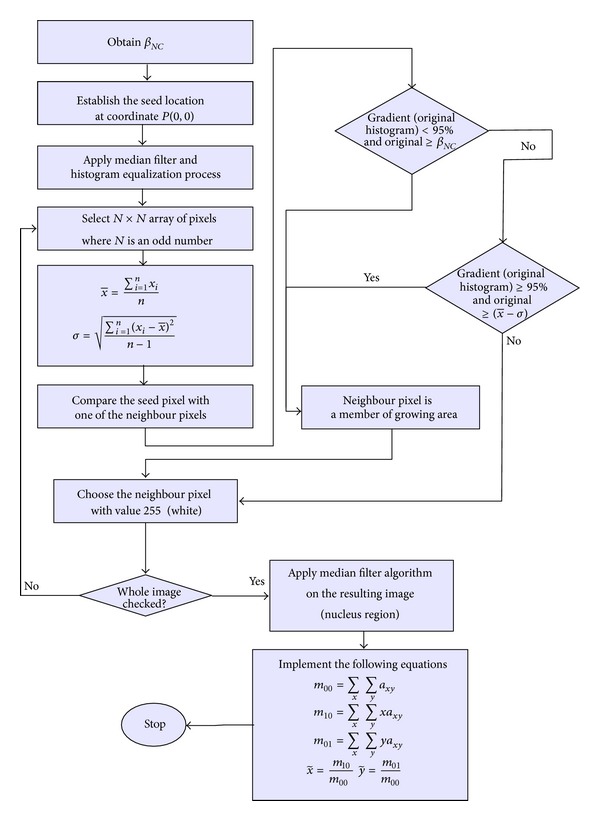
Flowchart summarising the steps of SBRG algorithm.

The subclustering process of the nucleus region is based on the conventional seed-based region growing (SBRG) algorithm. A minor modification is implemented to the SBRG algorithm, as compared to the original SBRG algorithm where the seed location for the region growing process is located at the background of the cervical cell, that is, at coordinate (0,0). The flowchart of subclustering process using SBRG algorithm is shown in Figure 4.

### 3.3. Segmentation and Features Extraction

There are several approaches that can be used to perform segmentation by using the region growing method. In this study, the modified SBRG algorithm is adopted [30]. The algorithm is used to segment the cervical cell image into three main regions: nucleus, cytoplasm, and background. Simultaneously, all the features needed from the image are extracted. The features are size, average grey level value, and perimeter for both the nucleus and cytoplasm, respectively. These features are usually used by pathologist for screening cervix cancer disease. The modified SBRG algorithm can be implemented as follows.

(1) Obtain the nucleus-cytoplasm threshold value, *β*_*NC*_, cytoplasm-background threshold value, *β*_*CB*_, and centroid location, $\left(\tilde{x},\tilde{y}\right)$, from the techniques discussed in Sections 3.1 and 3.2. The centroid is used as a starting point for the algorithm to execute.
(2) Set the following variables:
  (a) *nucleus* *size* = 1, *cytoplasm* *size* = 1,
  (b) *total grey level nucleus* = grey level value for seed pixel, *GL*_*N*_(*x*, *y*),
   
*total grey level cytoplasm* = grey level value for seed pixel, *GL*_*C*_(*x*, *y*).
(3) Apply median filter and histogram equalization process on the image.
(4) Select *N* × *N* array of pixels, where *N* is an odd number.
(5) Establish initial seed pixel location at coordinate $\left(\tilde{x},\tilde{y}\right)$.
(6) Calculate average grey level value, $\overset{-}{x}$, and standard deviation, *σ*, of the seed pixel(s) grown so far using
  (2) $$\begin{array}{c} \overset{-}{x}=\frac{\sum_{i=1}^{n}x_{i}}{n},\\ \sigma =\sqrt{\frac{\sum_{i=1}^{n}{\left(x_{i}-\overset{-}{x}\right)}^{2}}{n-1}.} \end{array}$$
(7) Compare the seed pixel with one of the neighbour pixels. The neighbour pixel is selected to become member of the growing area if one of the following conditions is fulfilled:
  (a) if the gradient between the original grey level value and the grey level value after histogram equalization process is less than 95% AND the original grey level value is equal or greater than the *β*_*NC*_ value (*β*_*CB*_ value for Step 12);
  (b) if the gradient between the original grey level value and the grey level value after histogram equalization process is same or larger than 95% AND the original grey level value is same or larger than the difference between the $\overset{-}{x}$ and the *σ*; that is, $\left(\overset{-}{x}-\sigma \right)$.
   If one of the conditions is fulfilled,
    (i) increase the variable *nucleus size* by 1 (for Step 12, increase the variable *cytoplasm size* by 1),
    (ii) perform summation of grey level value of the selected pixel with grey level value of previous selected pixels, ∑_*x*_∑_*y*_ *GL*_*N*_(*x*, *y*). Assign the result to variable *total grey level nucleus *(for Step 12, perform summation of grey level value of the selected pixel with grey level value of previous selected pixels, ∑_*x*_∑_*y*_ *GL*_*C*_(*x*, *y*). Assign the result to variable *total grey level cytoplasm*).
(8) Choose the neighbour pixel that has fulfilled the growing conditions as the new seed pixel.
(9) Mark the pixel as *Checked*.
(10) Assign the pixel with value 0 (black).
(11) Repeat Steps 5 to 9 by taking the neighbour pixel that has fulfilled the growing conditions as the new seed pixel until the entire image has been checked or cannot be grown. Mark the border pixel as *nucleus border.*
(12) Take *β*_*CB*_, and repeat Steps 5 to 7 for cytoplasm region by using the same centroid pixel, $\left(\tilde{x},\tilde{y}\right)$ as seed pixel.
(13) Assign the pixel that fulfilled the growing conditions and has not yet being marked as *Checked *with value 127 (grey).
(14) Repeat Steps 12 and 13 until the entire image has been examined or cannot be grown. Mark the border pixel as *cytoplasm border*.
(15) Mark all other pixels that are not involved in the growing process with value 255 (white).
(16) Count the pixel that has been marked as *nucleus border* and *cytoplasm border*. The perimeter of nucleus, *P*_*N*_, is equal to total amount of pixel marked with *nucleus border*, and the perimeter of cytoplasm, *P*_*C*_, is equal to total amount of pixel marked with *cytoplasm border*.
(17) The size of nucleus, *n*, is equal to value of *nucleus size*. For cytoplasm, the actual size, *c*, is equal to
  (3) $$\begin{array}{c} c=\text{cytoplasm}\;\text{size}-n. \end{array}$$
(18) Calculate the average grey level value for the nucleus, ${\bar{GL}}_{N}$, using
  (4) $$\begin{array}{c} {\bar{GL}}_{N}=\frac{\sum_{x}\sum_{y}{GL}_{N}\left(x,y\right)}{n}. \end{array}$$

Calculate the average grey level value for the cytoplasm, ${\bar{GL}}_{C}$, using

(5) $$\begin{array}{c} {\bar{GL}}_{C}=\frac{\sum_{x}\sum_{y}{GL}_{C}\left(x,y\right)-\sum_{x}\sum_{y}{GL}_{N}\left(x,y\right)}{C}. \end{array}$$

A few modifications have been applied on the conventional SBRG algorithm so that the modified SBRG algorithm is capable of automatically extract the features from the cervical cell image. Processes to obtain the threshold values and the initial seed location are carried out automatically. The threshold values, *β*_*NC*_ and *β*_*CB*_, are obtained from MKM clustering algorithm whereas the initial seed location is acquired from the invariant moment technique.

The modified SBRG algorithm is simultaneously used for segmentation and features extraction. Steps 10, 13, and 15 segment the image so that the nucleus, cytoplasm, and background region will be marked as black, grey, and white, respectively. Thus, the background region will be easily eliminated from the image.

The extraction process begins at Step 2 where the centroid is automatically accepted as nucleus region member. This is because the determination process for the centroid location is basically done using clustering technique in nucleus region. The neighbour pixels will then be examined and will be accepted as nucleus region member if the growing condition is fulfilled. It will then increase the number of pixels grown by one, according to Step 7. At the same time, the grey level value of the new member will be extracted and summed with total of grey level value of accepted members. After completing the growing process, the total number of nucleus region members will represent the size of nucleus, *n*, as shown in Step 17. Pixels that formed the border of nucleus will be calculated as perimeter of the nucleus, *P*_*N*_. The average grey level value for nucleus region is given by (4).

**Figure 5:**
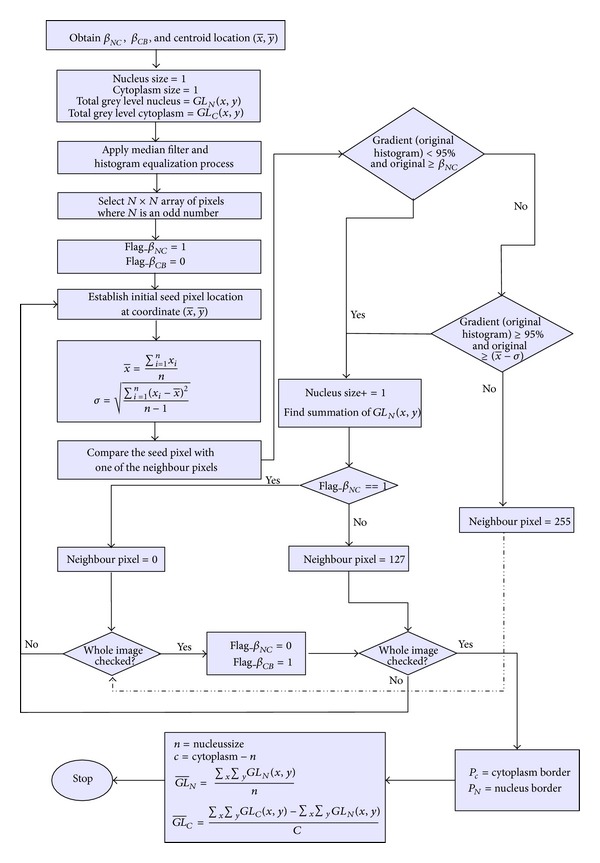
Flowchart summarising the steps of modified SBRG algorithm.

Region growing process for extracting cytoplasm features started at Step 12. By using the same methods, only pixels that fulfilled the conditions are accepted to become cytoplasm region members. The region growing process for the cytoplasm starts at the same seed location of the nucleus. Thus, the features extracted will contain the same features obtained from the extraction process of nucleus region, except for perimeter. Therefore, after completing the region growing process for the cytoplasm region, the number of pixels grown will be deducted with size of nucleus, *n*, to get the actual size of cytoplasm, *c*, as shown in (3). For the average grey level value, the summation of grey level for cytoplasm will be deducted with the summation of grey level for nucleus before dividing the result with the actual size of cytoplasm, *c*, as shown in (5).The flowchart of modified SBRG algorithm is presented in Figure 5.

## 4. Discussion of AFE Algorithm

**Figure 6:**
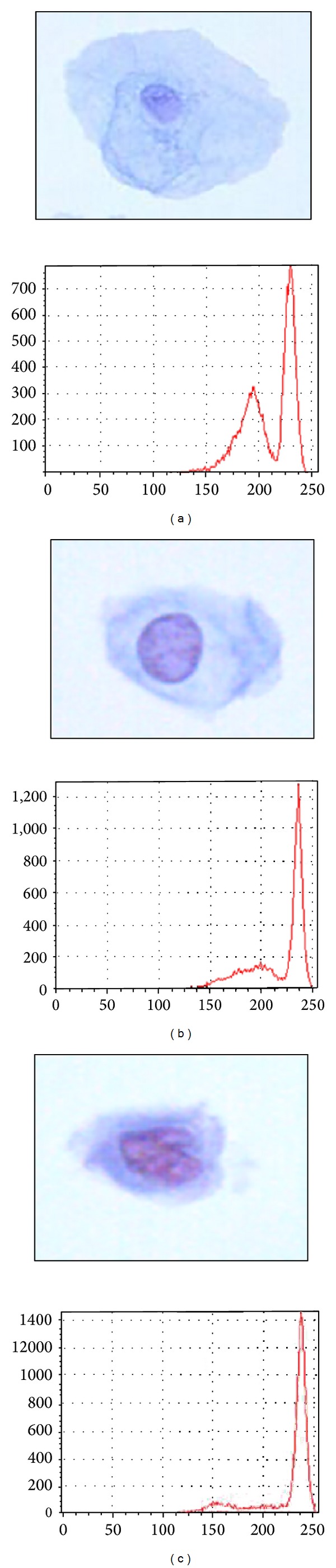
Cervical cancer image and grey level histogram.

The AFE algorithm is used to perform the extraction process of the cervical cell images. From 500 single cell images, 3 images (normal, LSIL, and HSIL) were selected arbitrarily for analysis in this section. For fair comparison, the resolution of every single image is saved as 160 × 120. Figures 6(a), 6(b), and 6(c) represent the normal, LSIL, and HSIL images with their grey level histogram, respectively. In the grey level histogram figure, the *x*-axis represents the grey level value, and the *y*-axis represents the number of pixels.

### 4.1. Threshold Values, *β*_*NC*_, and *β*_*CB*_

The first process that needs to be carried out is clustering process. The clustering process starts with histogram analysis to obtain the initial centres, *C*_*No*_, *C*_*Co*_, and *C*_*Bo*_ for nucleus, cytoplasm, and background region, respectively. The MKM clustering algorithm is then used to determine the final value of each centre that is referred to as *C*_*N*_, *C*_*C*_, and *C*_*B*_. The final centres values for each cervical cell image as tabulated in Table 1 are located at the correct location according to the grey level histogram in Figures 6(a), 6(b), and 6(c). The *C*_*N*_, *C*_*C*_, and *C*_*B*_ final centres are located at the centre of nucleus, cytoplasm, and background of their grey level histogram region, respectively. The final centre values were used to calculate the threshold values for nucleus-cytoplasm and cytoplasm-background region. To find all the members for each centre, the Euclidean distance concept was adopted. From the concept, to find the threshold value between nucleus and cytoplasm, *β*_*NC*_, and the threshold value between cytoplasm and background, *β*_*CB*_, the average value between *C*_*N*_ and *C*_*C*_ and the average value between *C*_*C*_ and *C*_*B*_ have been calculated, respectively. The results of final centre and threshold values for normal, LSIL, and HSIL image are presented in Table 1. From the table and its corresponding grey level histogram, the *β*_*NC*_ and *β*_*CB*_ are the grey level values which limit the group of pixels inside the nucleus and cytoplasm clusters, respectively. From the results, the threshold values have been successfully determined by using the MKM clustering algorithm.

### 4.2. Centroid Location, $\left(\tilde{x},\tilde{y}\right)$

**Figure 7:**
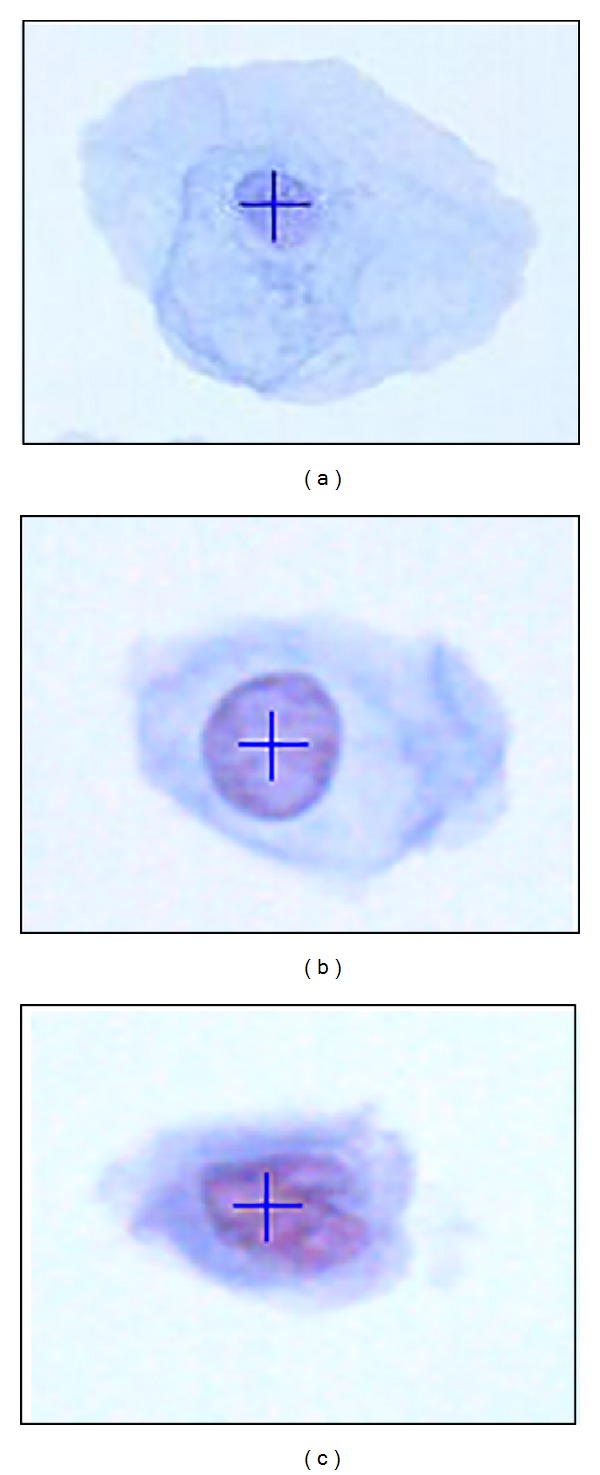
(a) Centroid location for normal, (b) LSIL, and (c) HSIL.

Centroid location finding is the second process after obtaining the threshold values, for the segmentation and features extraction process could start. The centroid is located inside the nucleus of the cervical cell and needs to be determined automatically. Thus, a process called subclustering is needed to distinguish the cytoplasm and background region of the cell. The *β*_*CB*_ values from the clustering process are used to subcluster the images. The nucleus area is used to find the centroid location by using the invariant moment technique. As shown in Figure 7, the centroid location is *x* = 72, *y* = 51 for normal cell, *x* = 71, *y* = 65 for LSIL, and *x* = 68, *y* = 56 for HSIL. From the observation, it can be noted that the centroid location for each image is located at the centre of the nucleus area. From the results, it can be stated that the process of finding the centroid location has been successfully achieved by using the subclustering and the invariant moment technique.

### 4.3. Segmentation

**Figure 8:**
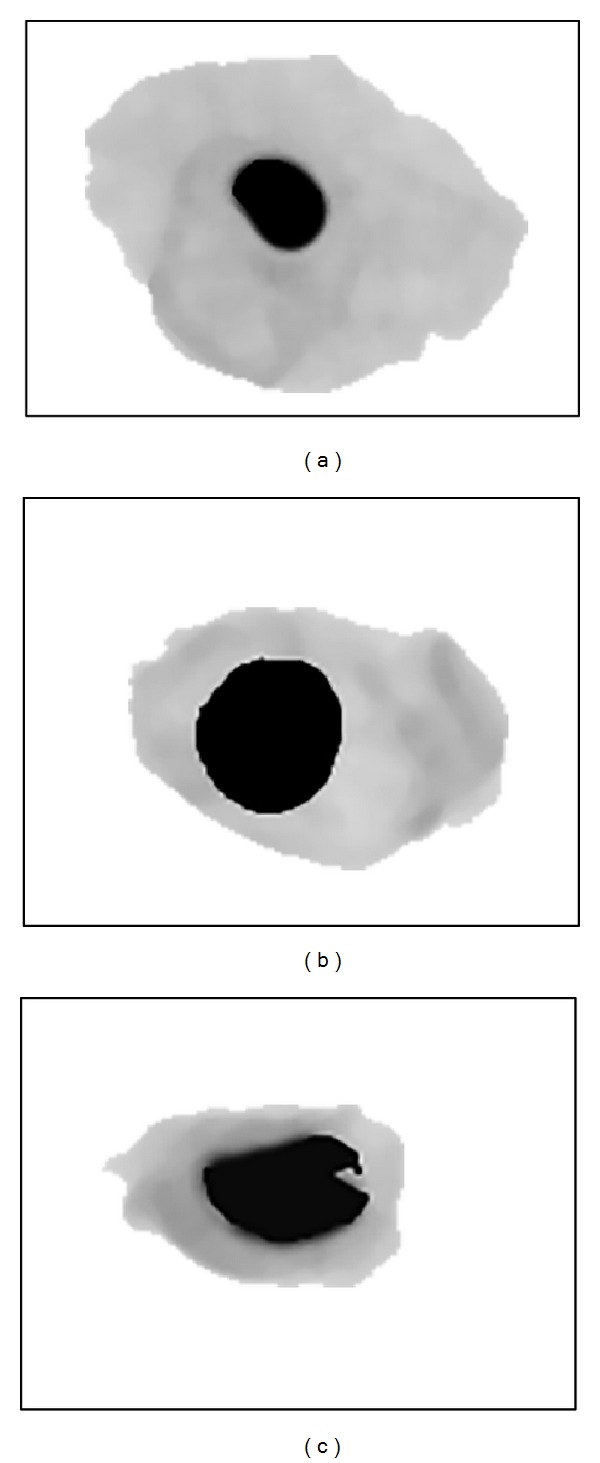
(a) Segmentation result for normal, (b) LSIL, and (c) HSIL.

After getting the threshold values and the centroid location of the cervical cell image, segmentation process is executed. The algorithm involved is modified SBRG algorithm that starts at centroid location determined earlier and grows towards the image border. Pixels values that are equal to or smaller than the *β*_*NC*_ value (and some other conditions described in growing condition, Section 3.3) are marked with value 0 indicating the nucleus region. For the cytoplasm region, pixels values that are equal to or smaller than the *β*_*CB*_ value (and some other conditions described in growing condition, Section 3.3) are marked with value 127. The remaining pixels values are assigned with value 255 for the background of the cervical cells. The results shown in Figure 8 proved that the modified SBRG algorithm has successfully segmented the cervical cell images.

### 4.4. Features Extraction

The final step in order to extract the features of the cervical cells is features extraction process. The process is executed simultaneously with the segmentation process presented in the previous section. Thus, the algorithm involved is modified SBRG algorithm that read the pixels value at the same time when the segmentation process is executed. The size and grey level value for nucleus and cytoplasm were updated each time a new pixel that belongs to nucleus and cytoplasm clusters was examined. Pixels that form a border between nucleus-cytoplasm and cytoplasm-background were marked and updated during the extraction process. The details about features extraction process were discussed in Section 3.3. The features that were extracted are shown in Table 2. The results showed that the normal cell has smaller nucleus area and a very large cytoplasmic area, whereas abnormal cell has increased nucleus area but shrinking cytoplasmic area.

## 5. Classification

The artificial neural network (ANN), fuzzy inference system (FIS), and neuro-fuzzy [34–47] are promising techniques which have proven to be very reliable in recent years. Neuro-fuzzy hybrid systems integrate the advantages of fuzzy systems for dealing with explicit knowledge which can be explained and understood and neural networks for dealing with implicit knowledge which can be acquired by learning. Therefore, combination of fuzzy system and neural networks handles limitations of both methods and offers an excellent data-mining opportunity to solve the critical and complex problem in pattern recognition [48]. One of most common tools is ANFIS which combined both fuzzy logic and neural network [49, 50].

**Figure 9:**
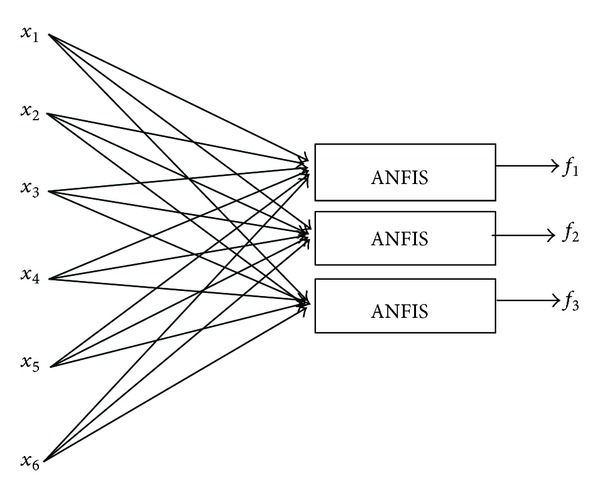
The architecture of the MANFIS with 6 inputs and 3 outputs.

The acronym ANFIS stands for adaptive neuro-fuzzy inference system which is a multi-input, single-output model [51]. However, A multioutput model can be designed by connecting few single output models. Thus, a multiple adaptive neuro-fuzzy inference system (MANFIS) is proposed in the presented study. The MANFIS contains a number of ANFIS models which are arranged in parallel combination to produce a model with multiple outputs.  Figure 9 shows an example of MANFIS with six inputs, *x*_1_, *x*_2_, *x*_3_, *x*_4_, *x*_5_, and *x*_6_, and three outputs, *f*_1_, *f*_2_, and *f*_3_. A hybrid learning algorithm which combines least squares estimation and back propagation is used for membership function parameter estimation. The advantage of hybrid method is that it uses back propagation for parameter associated with input membership function and least square estimation for parameters associated with output membership.

A total of 500 single cell images have been used in the classification process (376 normal, 79 LSIL, and 45 HSIL), where 80% of the cells (400 images) have been used for training and 20% of the cells (100 images) have been used for the MANFIS testing. Based on the analysis method which is proposed by Hoang [52], the fivefold analysis method is chosen. The accuracy percentage of each class is calculated by dividing the summation of the predicted number of cells over the summation of the original number of samples in each class in all folds. The results obtained from the five tests are then averaged [53]:

(6) Accuracy=[Number  of  correctly  classified  samplesTotal  cell  samples] ∗100%.

Table 3 shows the number of samples of each cell category randomly selected for each fold. The results that have been yielded by the MANFIS in each fold are illustrated in Table 4. The table presents the number of predicted samples for each category of cell class (i.e., the normal, LSIL, and HSIL). The normal cells attain highest accuracy (97.3%) as compared to the LSIL (92.6%) and HSIL (93.7%) because the HSIL is almost similar to the LSIL. The average accuracy is measured based upon (6), and the produced results for overall classification process are 96.3% and 94.2% for training and testing phases, respectively. Thus, with the high accuracy obtained from the classification process, the system is concluded to be reliable and suitable to be applied for cervical cancer classification.

## 6. Conclusion

An intelligent computer vision system has been developed for the classification of cervical cancer based on its morphological cell characteristics. The developed system is able to categorize the cervical cells into three groups, namely, normal, LSIL, and HSIL. The system consists of AFE algorithm and MANFIS classifier. The AFE algorithm is started with clustering technique using moving *k*-mean algorithm to find the threshold value to differentiate nucleus, cytoplasm, and background area of the cervical cell image. Invariant moment technique is performed to get the centroid or seed location for features extraction process. Segmentation and features extraction are then executed using modified SBRG technique. The AFE algorithm is capable to extract 6 features from the images (size, perimeter, and average grey level for both nucleus and cytoplasm). Finally, the features are used as inputs to the MANFIS for classification of the type of cervical cells. The training accuracy produced by MANFIS was 96.3% and the testing accuracy was 94.2% based on the fivefold analysis method. The system is proven to be very efficient and also helpful to be used by medical lab technicians and biologists. As it is desired to have high performance, improvement can be made by including other parameters for classification like nucleus to cytoplasm ratio, saturation, intensity, and so forth.

## Figures and Tables

**Table 1:** Final centres and threshold values for normal, LSIL, and HSIL.

Image	Final centres			Threshold values	
	*C*_*N*_	*C*_*C*_	*C*_*B*_	*β*_*NC*_	*β*_*CB*_
Normal	153	190	227	171	208
LSIL	161	194	234	177	214
HSIL	152	187	235	169	211

**Table 2:** Features extracted from normal, LSIL, and HSIL.

Image	Cells'	Features		
		Size	Perimeter	Gray level
		(pixels^2^)	(pixels)	(0–255)
Normal	Nucleus	337	89	145.875
	Cytoplasm	8859	1282	187.512
LSIL	Nucleus	1155	307	160.565
	Cytoplasm	6239	550	186.131
HSIL	Nucleus	1327	320	151.455
	Cytoplasm	3675	373	173.084

**Table 3:** The original number of the cervical cell images classes for the fivefold method.

	Training samples (80%)			Testing samples (20%)		
	Normal	LSIL	HSIL	Normal	LSIL	HSIL
Fold 1	300	64	36	76	15	9
Fold 2	310	62	28	66	17	17
Fold 3	308	58	34	68	21	11
Fold 4	317	52	31	59	27	14
Fold 5	309	62	29	67	17	16

**Table 4:** The predicted number of the cervical cell images classes for the fivefold method and the accuracy produced for each category and the overall accuracy of the training and test phases.

	Training			Testing			Accuracy	
	Normal	LSIL	HSIL	Normal	LSIL	HSIL	Acc. train	Acc. test
Fold 1	294	62	35	73	13	8	97.8	94.0
Fold 2	304	56	25	61	15	16	96.3	92.0
Fold 3	298	51	32	65	20	10	95.3	95.0
Fold 4	306	48	29	54	26	13	95.8	93.0
Fold 5	300	59	27	66	16	15	96.5	97.0

Average accuracy	97.3	92.6	93.7	94.9	92.8	92.5	96.3	94.2
